# Factors Affecting Clinical over and Underestimation of Fetal Weight—A Retrospective Cohort

**DOI:** 10.3390/jcm11226760

**Published:** 2022-11-15

**Authors:** Gal Cohen, Hila Shalev-Ram, Hanoch Schreiber, Omer Weitzner, Yair Daykan, Michal Kovo, Tal Biron-Shental

**Affiliations:** 1 Department of Obstetrics and Gynecology, Meir Medical Center, 59 Tchernichovsky St., Kfar Saba 44281, Israel; 2 Sackler School of Medicine, Tel Aviv University, Tel Aviv 39040, Israel

**Keywords:** overestimation of fetal birthweight, underestimation of fetal birthweight, clinical estimation of fetal birthweight, inaccuracy in fetal weight estimation, inaccurate fetal weight estimation

## Abstract

Clinical estimation of fetal weight is an integral component of obstetric care that might dictate the timing and mode of delivery. Inaccurate fetal weight estimation might result in unnecessary interventions or in underestimating potential risks, resulting in inappropriate intrapartum care. This retrospective study assessed factors associated with under- or overestimation of birthweight and evaluated the obstetric implications. It included singleton births ≥24 w with clinically estimated fetal weight (EFW) up to 1 week before delivery, during 2014–2020. Estimates >±10% of the actual birthweight were considered inaccurate and categorized as overestimation (>10% heavier than the actual birthweight) or underestimation (>10% smaller than the birthweight). Multivariable logistic regression was performed to reveal factors associated with inaccurate EFW. Maternal characteristics and obstetric outcomes were compared. The primary outcomes for the overestimation group were the neonatal composite adverse outcome, induction of labor and cesarean delivery rates. The primary outcomes for the underestimation group were rates of shoulder dystocia, 3rd- or 4th-degree perineal lacerations, and failed vacuum extraction. Among 38,615 EFW, 5172 (13.4%) were underestimated, 6695 (17.3%) were overestimated and 27,648 (69.3%) accurate. Multivariable logistic regression found increasing gestational age as an independent risk-factor for underestimation (odds ratio (OR) 1.15 for every additional week, 95% confidence interval (CI) 1.12–1.2). Major factors independently associated with overestimation were nulliparity (OR 1.95, CI 1.76–2.16), maternal obesity (OR 1.52, CI 1.33–1.74), smoking (OR 1.6, CI 1.33–1.93), and oligohydramnios (OR 1.92, CI 1.47–2.5). Underestimation was an independent risk-factor for shoulder dystocia (OR 1.61, CI 1.05–2.46) and 3rd- or 4th-degree perineal lacerations (OR 1.59, CI 1.05–2.43). Overestimation was an independent risk-factor for neonatal composite adverse outcome (OR 1.15, CI 1.02–1.3), induced labor (OR 1.30, CI 1.21–1.40) and cesarean delivery (OR 1.59, CI 1.41–1.79). Clinicians should be aware of factors and adverse obstetric implications associated with over- or underestimation of birthweight.

## 1. Introduction

Fetal weight estimation is an integral component of obstetric care. Detecting the range of fetal weight is crucial for delivery planning, as it determines delivery timing, desired fetal monitoring, clinical interventions before and during delivery, required expertise of the accompanying medical staff, and at times, the mode of delivery [1,2,3,4,5,6]. Inaccurate fetal weight estimation might result in unnecessary interventions or in underestimating potential risks, resulting in inappropriate intrapartum care.

Clinically estimated fetal birth weight (EFW) is performed routinely at admission for delivery. It is simple, cost-effective and sometimes the only modality used to estimate fetal weight before delivery due to lack of medical staff and equipment. Sonographic EFW has been reported to be superior to EFW [7,8], especially for overweight women [9], for tall women [10] and for the lower range of birthweights (BW) (<2500 g) [11]. However, the contribution of ultrasound added to routine EFW in cases of suspected macrosomia remains unclear and might even increase the rates of unnecessary cesarean deliveries (CD) [12,13]. Previous studies evaluating whether the precision of EFW improves with the examiner’s experience were inconclusive [13,14]. As for the effect of amniotic fluid volume on EFW, a previous study concluded that it might be a confounding factor, even when not necessarily regarded as oligohydramnios or polyhydramnios [14].

Data regarding the clinical factors associated with inaccurate EFW are sparse and larger cohorts are needed to evaluate this question. Moreover, previous studies have described factors associated with inaccurate EFW per se, without evaluating under- and overestimations separately; thereby, leaving the question of specific clinical factors associated with each, unresolved.

Both low and high BW are associated with maternal and neonatal morbidity [15,16,17]. Since many clinical decisions during delivery are based on EFW rather than the actual BW, significant errors in these estimations are likely to result in adverse obstetric outcomes. However, none of the studies to date have evaluated the obstetric implications of inaccurate EFW.

This study assessed clinical factors associated with over- and underestimations of BW, performing an EFW, and evaluated the obstetric implications. Our hypothesis was that overestimation will result in unnecessary interventions due to suspected macrosomia, and will, on the other hand, increase the risk for neonatal adverse outcomes due to underdiagnosis of low BW neonates and inappropriate intrapartum care.

We also hypnotized that underestimation of BW will increase the risk for obstetric adverse outcomes associated with macrosomia, due to underdiagnosis of marcosomic neonates and inappropriate management during delivery.

## 2. Methods

This retrospective cohort study included live singleton births at 25+ weeks gestational age (GA), delivered in a tertiary care medical center in Kfar Saba, Israel from January 2014 to September 2020. Multiple pregnancies, cases of intrauterine fetal demise and deliveries without a valid clinical EFW at admission or within one week before delivery were excluded. Physicians used the Leopold maneuver to estimate fetal weight [17]. Deviations more than ±10% of the actual BW were defined as inaccurate and categorized as overestimation (>10% heavier than the actual BW) or underestimation (>10% smaller than the BW). The medical records of the 3 EFW groups were compared in terms of basic maternal factors, labor and delivery characteristics, as well as maternal and neonatal outcomes.

Univariate analysis and multivariable logistic regression were performed to reveal factors associated with over- or underestimation of BW. Maternal and neonatal outcomes were compared according to EFW.

The primary outcomes for the overestimation group were the neonatal composite adverse outcome of hypoglycemia, hypothermia, meconium aspiration, need for non-invasive ventilation or phototherapy, and induction of labor (IOL) and cesarean delivery (CD) rates. The primary outcomes for the underestimation group were rates of shoulder dystocia, 3rd- or 4th-degree perineal lacerations, and failed vacuum extraction (VE).

### 2.1. Data Collection

Data were retrieved using the electronic database of the obstetric triage unit and the delivery room, which were cross-tabulated with data from the neonatal unit and the neonatal intensive care unit. Medical records were reviewed by the principal investigator to complete missing data.

The following information was collected from the patients’ electronic medical records:

1. Maternal demographics and medical history (age, gravidity, parity, height, weight, body mass index (BMI, kg/m^2^), maternal stature (short: height <10th percentile or tall: >90th percentile), rates of obesity (BMI > 30 kg/m^2^), smoking, chronic hypertension, pregestational diabetes mellitus (DM), gestational diabetes mellitus (GDM) [18], prior preterm deliveries, prior first or second trimester abortions and background morbidities).
2. Data at presentation, including clinical and sonographic EFW [19], premature uterine contractions, preterm or premature rupture of membranes, vaginal bleeding, placental implantation site, fetal presentation, preeclampsia or gestational hypertension diagnosed according to international guidelines [20] and rates of polyhydramnios or oligohydramnios [21].
3. Delivery characteristics: GA at delivery, onset of delivery (spontaneous/induced), use of epidural anesthesia, intrapartum fever (a measurement of maternal fever ≥38 degree Celsius during delivery or up to 24 h from delivery), amniotic fluid color, fetal sex, mode of delivery (normal vaginal delivery, VE, CD), indication for VE or CD and intrapartum maternal blood loss.
4. All neonates were evaluated by a pediatrician immediately after delivery, or by a neonatologist if the birthweight was <2500 g. Neonatal outcome data collected were Apgar scores, umbilical cord pH, fetal macrosomia, neonatal birthweight (small for gestational age (SGA), average for gestational age (AGA) or large for gestational age (LGA), diagnosed according to local birth weight charts [22]. Neonatal diagnoses were determined by the pediatrician at delivery and during neonatal hospitalization, according to international standards, relevant blood samples and imaging.

### 2.2. Ethics Approval

The study was approved by the Meir Medical Center Ethics Committee in September 2021, approval number 0167-21-MMC. Due to the retrospective nature of the data collection, individual informed consent was waived by the Ethics Committee.

### 2.3. Statistical Analysis

Categorical data were compared using Chi-square or Fisher’s exact test, each when appropriate. Continuous variables between groups were compared using *t*-test. Multivariable logistic regression and adjusted odds ratios (OR) were calculated to examine independent risk-factors for over or underestimation of neonatal BW and for the primary outcomes, after adjusting for potential confounders. A probability value of <0.05 was considered significant. All analyses were performed using SPSS-26 software (IBM Corp., Armonk, NY, USA). Due to the strict protocol and detailed documentation in our institution, the rate of missing data in the final study cohort was negligible (~0.01%) and was not needed to be further addressed.

## 3. Results

**Figure 1:**
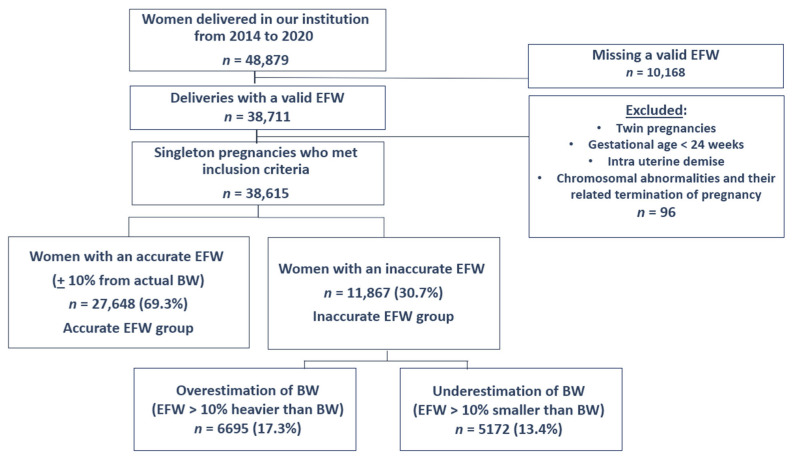
Flowchart describing the study population.

**Figure 2:**
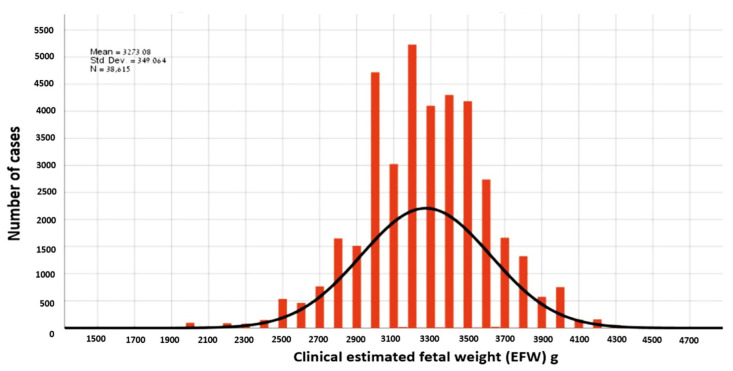
Distribution of clinical estimated fetal weight.

**Figure 3:**
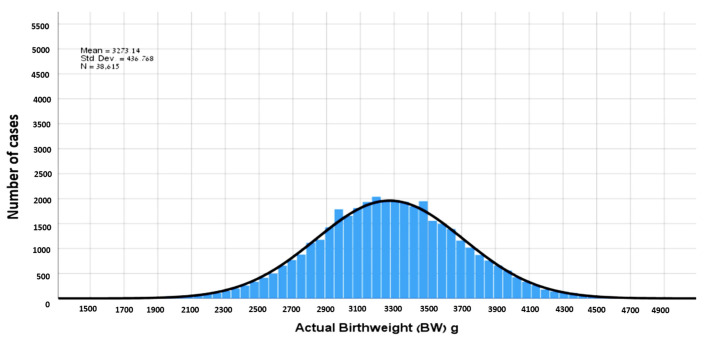
Distribution of actual birthweights.

During the study period, 48,879 women delivered in our institution, of which 38,615 had a singleton pregnancy with a valid EFW at admission and met the inclusion criteria (Figure 1). The distribution of EFW and the actual BW in our cohort is presented in Figure 2 and Figure 3. The cohort included 6695 (17.3%) women with an EFW more than 10% heavier than the neonatal actual BW (overestimation group), 5172 (13.4%) women with EFW more than 10% smaller less than the actual BW (underestimation group) and 27,648 (69.3%) women with an EFW that was within 10% of the actual BW (accurate EFW group).

### 3.1. Univariate Analysis

Table 1 and Table 2 present the maternal, delivery and neonatal characteristics of the overestimation and underestimation groups, respectively compared to the accurate EFW group.

### 3.2. Multivariable Logistic Regression Analysis

Risk-factors for over- or underestimation of BW.

After multivariable logistic regression analysis, the following factors were found to be independently associated with overestimation: nulliparity (adjusted odds ratio (OR) 1.95, 95% CI 1.76–2.16), maternal obesity (OR 1.52, CI 1.33–1.74), maternal short stature (OR 1.35, CI 1.17–1.56), smoking (OR 1.6, CI 1.33–1.93), hypertensive disorders (OR 1.3, CI 1.03–1.63), oligohydramnios (OR 1.92, CI 1.47–2.5) and male fetus (OR 1.44, CI 1.3–1.58).

Increasing GA at delivery was found to be an independent risk-factor for underestimating weight (OR 1.15, 95%CI 1.12–1.2 for every additional week). Table 3 presents the results of the multivariable logistic regression analysis of clinical factors associated with over- or underestimation of BW.

### 3.3. Obstetric Implications of over or Underestimation

The multivariable regression model revealed that overestimation of neonatal BW was an independent risk-factor for neonatal composite adverse outcome (OR 1.15, CI 1.02–1.3), when adjusted for nulliparity, GA at delivery and neonatal BW. Overestimation was also independently associated with higher rates of both IOL and CD (OR 1.30, CI 1.21–1.40 and OR 1.59, CI 1.41–1.79, respectively), when adjusted for nulliparity, GA at delivery, maternal obesity, diabetes mellitus, hypertensive disorders and neonatal BW.

Underestimation of neonatal BW was an independent risk-factor for shoulder dystocia (OR 1.61, CI 1.05–2.46) and 3rd-or 4th-degree perineal lacerations (OR 1.59, CI 1.05–2.43), when adjusted for nulliparity, maternal obesity, neonatal macrosomia and diabetes mellitus. Underestimation was not associated with increased risk for failed VE (Table 4).

### 3.4. The Effect of BW Itself on EFW

SGA neonates were more common in the overestimation group compared to the accurate EFW group (27.8% vs. 3.5%, *p* < 0.001). In contrast, macrosomic neonates were more common in the underestimation group (18.6% vs. 3.4% in the accurate EFW group, *p* < 0.001).

BW was not used in the multivariable logistic regression analysis evaluating risk-factors for inaccuracy, since this factor is only available after delivery.

### 3.5. Misclassification Due to over-and Underestimation of Neonatal BW

Overestimation created an underdiagnosis of SGA neonates: In the overestimation group, 1862 (27.8%) neonates were truly SGA. Of them, 1768 (95.0%) were misclassified as AGA. Overestimation also misdiagnosed LGA neonates, creating false positive rates of LGA fetuses: In the overestimation group, only 26 (0.4%) neonates were truly LGA, but a total of 546 (8.2%) were classified as LGA, meaning 517 (10.8%) of the AGA fetuses were misclassified as LGA.

Underestimation created an underdiagnosis of LGA neonates: In the underestimation group, 1524 (29.4%) neonates were truly LGA. Of them 1442 (94.6%) were misclassified as AGA. Underestimation also misdiagnosed SGA neonates, creating false positive rates of SGA fetuses: In the underestimation group, only 26 (0.5%) were truly SGA, but a total of 520 (10.1%) were classified as SGA, meaning 491 (13.6%) of the AGA fetuses were misclassified as SGA.

## 4. Discussion

This study summarized the clinical factors associated with over- and underestimation of BW when performing an EFW, as well as the obstetric implications. It is the first large cohort study to differentiate overestimation from underestimation and investigate the specific risk-factors associated with each error. Main findings indicate that nulliparity, maternal obesity, maternal short stature, smoking, hypertensive disorders, oligohydramnios and a male fetus were independently associated with overestimated BW, while increasing GA at delivery was independently associated with underestimated BW.

Inaccurate fetal weight estimation had adverse obstetric implications. Overestimation was an independent risk-factor for the neonatal composite adverse outcome, IOL and CD. Underestimation was an independent risk-factor for both shoulder dystocia and 3rd- or 4th-degree perineal lacerations.

This study revealed that maternal obesity is an independent risk-factor for overestimation and a protective factor from underestimation. Maternal obesity was previously described as a risk-factor for inaccurate clinical EFW compared to sonographic EFW, with a significantly higher absolute error using clinical EFW among overweight women [10] Farrel et al., revealed higher rates of underestimation in low BMI patients and higher rates of overestimation in high BMI patients [23]. Our findings agree with these data and indicate that the inaccuracy related to obesity is mainly overestimation.

We found that oligohydramnios was an independent risk-factor for overestimation. Data regarding the association between oligohydramnios and inaccurate estimation of neonatal BW were previously described mainly for sonographic EFW [24]. Yet, our findings are in agreement with a previous study in which clinical EFW were influenced by the placental size and the fluid volume [14]. Of note, this study excluded patients with oligohydramnios or polyhydramnios. In our cohort, anterior placentas were more common in the overestimation group; however, the effect was not significant in multivariable analysis. This finding might be because it is technically difficult to evaluate fetal size apart from the placenta when it is located in the anterior portion of the uterus.

Interestingly, although previous reports found discrepancies between clinical compared to sonographic EFW among tall women, concluding that clinical EFW is less accurate among tall women [11], the current study did not reveal tall stature as a risk-factor for over nor underestimated BW. However, short maternal stature was a risk-factor for overestimated BW. These differences might be explained by evaluating overestimation and underestimation separately, which revealed the effect of maternal height on EFW more clearly.

It is logical that nulliparity is a risk-factor for inaccurate EFW, because there is no previous BW for comparison, which might improve the current EFW [25]. Even so, previous studies comparing nulliparous and multiparous patients found that they were equally accurate in estimating neonatal BW [26]. We found that nulliparity is a specific risk-factor for overestimation and a protective factor from underestimation. This finding supports the concept of evaluating the ramifications of over- and underestimation separately.

When assessing the influence of BW alone, we found that physicians tend to estimate the fetal weight closer to the average, with more SGA neonates in the overestimation group and more macrosomic neonates in the underestimation group. This was also reflected by high rates of misclassification of SGA neonates as AGA in the overestimation group, and by high rates of misclassification of LGA neonates as AGA in the underestimation group. These findings were described previously [8,27] and make sense since an EFW far from the average BW might cause self-doubt, subconsciously making the physician revise the evaluation closer to the average neonatal BW.

Older GA at delivery was previously found to decrease the accuracy of both clinical and sonographic EFW, with a linear regression analysis showing decreased precision with increasing GA [8]. We found that as GA increases, the risk for underestimation increases, while the risk for overestimation decreases. These findings were demonstrated in the univariate analysis, which showed higher rates of late-preterm infants and lower rates of post-term pregnancies in the overestimation group, as well as in the multivariable analysis. These findings might also imply that physicians tend to estimate the fetal weight closer to the expected weight based on GA.

Smoking and hypertensive disorders were risk-factors for overestimation. These findings have not been reported previously, and might be the result of the lower neonatal BW associated with both factors, and physicians’ tendency to overestimate smaller neonates.

The association between macrosomia, shoulder dystocia and 3rd- or 4th-degree perineal laceration is well-established [16,27,28]. However, the current study is the first to reveal that underestimating BW, regardless of the actual BW, increases the risk for both outcomes. These findings might be explained by lack of awareness of the high BW, resulting in a lack of the appropriate preparations which are known to reduce the risk for these adverse outcomes during delivery (lithotomy position, presence of a skilled obstetrician during delivery, protective episiotomy, avoidance of operative vaginal delivery, etc.) [27,29].

The association between low BW and adverse neonatal outcomes has also been described previously [15,30]. However, our findings demonstrate that overestimating the BW resulted in higher rates of the composite neonatal adverse outcome, regardless of actual BW or GA at delivery. We believe these findings were the result of not anticipating the neonate’s relatively minimal reserves and reduced ability to cope with intrapartum stress.

Another novel aspect of this study is demonstrating that overestimating an AGA neonate might expose the mother to unnecessary interventions, as overestimation was found an independent risk-factor for IOL and for CD in our cohort. These implications were previously reported only for inaccurate sonographic EFW [31] and make sense given the ACOG guidelines recommending CD for women with an EFW >5000 g or >4500 g for mothers with diabetes [32], and previous reports showing better outcomes for macrosomic neonates after IOL at 37 to 38 6/7 weeks of gestation compared to expectant management [33].

### 4.1. Clinical Implications

Clinical fetal weight estimation is a simple and cost-effective modality used world-wide. Our findings indicate that inaccurate EFW exposes the mother and neonate to adverse outcomes. Physicians should strive to improve their estimations of fetal weight to improve maternal and neonatal outcomes. Several clinical factors are associated with overestimation or underestimation of the actual BW and physicians should be more aware of the different clinical factors associated with each.

### 4.2. Strengths and Limitations

The strengths of this study include the large cohort and detailed documentation that allowed analysis of a large amount of clinical data and assembling a novel detailed profile of the risk-factors associated with over- or underestimation of neonatal BW based on EFW. Data were retrieved from a single institution with the same medical protocols and diagnostic tools, creating a relatively homogenous cohort. This is the first study differentiating overestimation from underestimation, and specifying the risk-actors and obstetric implications related to each.

Limitations to this study include its retrospective design, which resulted in some missing data, including long-term maternal and neonatal outcomes. Therefore, only short-term outcomes were analyzed. Sonographic EFW was not performed routinely; thus, its possible influence on the physician’s clinical EFW is unclear. Inter-observer variability in fetal weight estimations is possible depending on physician’s experience. However, the study included both new and experienced physicians; thus, we believe it faithfully represented the variety of staff.

## 5. Conclusions

We found that nulliparity, maternal obesity, maternal short stature, smoking, hypertensive disorders, oligohydramnios and a male fetus were associated with overestimated BW, while increasing GA at delivery was associated with underestimated BW. Overestimation was found a risk-factor for the neonatal composite adverse outcome, IOL and CD, while underestimation was an independent risk-factor for both shoulder dystocia and 3rd- or 4th-degree perineal lacerations. Clinicians should be more aware of the clinical factors and adverse obstetric implications associated with over- or underestimating neonatal BW when performing a clinical EFW, and strive to improve their estimations.

## Figures and Tables

**Table 1:** Maternal, delivery and neonatal characteristics of the overestimation group compared to the accurate EFW group.

Variable		Estimated Birthweight		
		Overestimation (*n* = 6695)	Accurate (*n* = 27,648)	*p*-Value
Maternal age (years), mean + SD		31.0 ± 5.5	31.0 ± 5.3	0.908
Gestational age at delivery (weeks), mean + SD		38.8 ± 1.4	39.3 ± 1.3	<0.001
Gestational week at delivery, per week (*n*, %)	<34 w	17 (0.3%)	44 (0.2%)	0.125
	34–36 + 6 w	327 (4.9%)	704 (2.6%)	<0.001
	≥37 w	6351 (94.9%)	26,000 (97.2%)	<0.001
	≥41 w	657 (9.8%)	3899 (14.6%)	<0.001
Maternal BMI, kg/m^2^ (mean + SD)		24.3 ± 4.0	23.9 ± 3.7	<0.001
Obesity (BMI > 30) (*n*, %)		567 (16.3%)	1670 (11.5%)	<0.001
Maternal weight gain during pregnancy, kg, (mean + SD)		11.87 ± 5.9	12.26 ± 6.0	<0.001
Maternal height, m (mean + SD)		1.63 ± 0.05	1.63 ± 0.05	<0.001
Maternal height under 10th percentile (*n*, %)		624 (9.3%)	2171 (8.1%)	0.001
Maternal height above 90th percentile (*n*, %)		554 (8.1%)	2257 (8.4%)	0.213
Smoker (*n*, %)		540 (8.1%)	1392 (5.2%)	<0.001
Nulliparity (*n*, %)		3183 (47.5%)	9353 (35.0%)	<0.001
Previous cesarean delivery (among multiparas (*n*, %))		634 (18.4%)	2340 (13.5%)	<0.001
Gestational DM/DM (*n*, %)		806 (12.0%)	2926 (10.9%)	0.011
Preeclampsia/Hypertension (*n*, %)		325 (4.9%)	741 (2.8%)	<0.001
Placental location (*n*, %)	Anterior	2167 (54.6%)	8520 (51.8%)	0.001
	Posterior	1266 (31.9%)	5743 (34.9%)	<0.001
	Previa/low lying	11 (0.3%)	45 (0.3%)	0.967
	Other	522 (13.2%)	2141 (13.0%)	0.807
Fetal sex (*n*, %)	Male	2902 (43.3%)	13,608 (50.9%)	<0.001
	Female	3793 (56.7%)	13,140 (49.1%)	
Fetal presentation (*n*, %)	Vertex	6084 (97.5%)	24,480 (98.6%)	<0.001
	Breech	147 (2.4%)	309 (1.2%)	<0.001
	Transverse	6 (0.1%)	27 (0.1%)	0.785
Polyhydramnios (*n*, %)		34 (0.5%)	103 (0.4%)	0.158
Oligohydramnios (*n*, %)		239 (3.6%)	560 (2.1%)	<0.001
Neonatal birth weight, mean + SD		2838.5 ± 330.7	3307.9 ± 374.4	<0.001
Small for gestational age (*n*, %)		1862 (27.8%)	934 (3.5%)	<0.001
Large for gestational age (*n*, %)		26 (0.4%)	2084 (7.8%)	<0.001
Macrosomia (*n*, %)		3 (0.0%)	914 (3.4%)	<0.001
Umbilical cord pH < 7 (*n*, %)		26 (1.2%)	80 (1.2%)	0.866
Umbilical cord pH < 7.1 (*n*, %)		122 (5.4%)	318 (4.8%)	0.218
5 min Apgar score <7 (*n*, %)		22 (0.3%)	91 (0.3%)	0.884
Neonatal composite outcome (Including hypoglycemia, hypothermia, meconium aspiration, non-invasive ventilation, phototherapy), (*n*, %)		499 (9.2%)	1543 (6.9%)	<0.001

**Table 2:** Maternal, delivery and neonatal characteristics of the underestimation group compared to the accurate EFW group.

		Estimated Neonatal Birthweight		
Variable		Underestimated (*n* = 5172)	Accurate (*n* = 27,648)	*p*-Value
Maternal Age (years) mean + SD		30.5 ± 5.2	31.0 ± 5.3	<0.001
Gestational age at delivery (weeks) mean + SD		39.5 ± 1.3	39.3 ± 1.3	<0.001
Gestational week at delivery, per week (*n*, %)	<34 w	12 (0.2%)	44, (0.2%)	0.288
	34–36 + 6 w	136 (2.6%)	704 (2.6%)	0.992
	≥37 w	5024 (97.1%)	26,000 (97.2%)	0.795
	≥41 w	968 (18.7%)	3899 (14.6%)	<0.001
Maternal BMI, kg/m^2^ (mean + SD)		23.3 ± 3.3	23.9 ± 3.7	<0.001
Obesity (BMI > 30) (*n*, %)		204 (7.4%)	1670 (11.5%)	<0.001
Maternal weight gain during pregnancy, kg (mean + SD)		12.47 ± 6.2	12.26 ± 6.0	0.021
Maternal height, m (mean + SD)		1.63 ± 0.06	1.63 ± 0.05	0.036
Maternal height under 10th percentile (*n*, %)		404 (7.8%)	2171 (8.1%)	0.461
Maternal height above 90th percentile (*n*, %)		464 (9.0%)	2257 (8.4%)	0.209
Smoking (*n*, %)		200 (3.9%)	1392 (5.2%)	<0.001
Nulliparity (*n*, %)		1410 (27.3%)	9353 (35.0%)	<0.001
Previous cesarean delivery (among multiparas (*n*, %)		356 (9.5%)	2340 (13.5%)	<0.001
Gestational DM/DM (*n*, %)		372 (7.2%)	2926 (10.9%)	<0.001
PET/HTN (*n*, %)		124 (2.4%)	741 (2.8%)	0.134
Placental location (*n*, %)	Anterior	1602 (51.1%)	8520 (51.8%)	0.492
	Posterior	1136 (36.3%)	5743 (34.9%)	0.148
	Previa/low lying	4 (0.1%)	45 (0.3%)	0.134
	Other	391 (12.5%)	2141 (13.0%)	0.413
Fetal sex (*n*, %)	Male	3127 (60.5%)	13,608 (50.9%)	<0.001
	Female	2045 (39.5%)	13,140 (49.1%)	
Presentation (*n*, %)	Vertex	4309 (98.4%)	24,480 (98.6%)	0.132
	Breech	65 (1.4%)	309 (1.2%)	0.236
	Transverse	7 (0.1%)	27 (0.1%)	0.362
Polyhydramnios (*n*, %)		14 (0.3%)	103 (0.4%)	0.214
Oligohydramnios (*n*, %)		82 (1.6%)	560 (2.1%)	0.018
Neonatal birth weight, mean + SD		3656.0 ± 400.3	3307.9 ± 374.4	<0.001
SGA (*n*, %)		26 (0.5%)	934 (3.5%)	<0.001
LGA (*n*, %)		1524 (29.5%)	2084 (7.8%)	<0.001
Macrosomia (*n*, %)		964 (18.6%)	914 (3.4%)	<0.001
3-4th degree perineal laceration (*n*, %)		59 (1.1%)	192 (0.7%)	0.002
Failed vacuum extraction (*n*, %)		22 (0.4%)	68 (0.3%)	0.034
Shoulder dystocia (*n*, %)		70 (1.4%)	163 (0.6%)	<0.001

**Table 3:** Multivariable logistic regression analysis—clinical factors associated with overestimation or underestimation of neonatal birthweight.

Factors Associated with Overestimation of Neonatal Birthweight *			
	Adjusted Odds Ratio	95% CI	*p* Value
Nulliparity	1.95	1.76–2.16	<0.001
Obesity (BMI > 30 kg/m^2^)	1.52	1.33–1.74	<0.001
Short stature (Maternal height below 10th percentile)	1.35	1.17–1.56	<0.001
Smoking	1.60	1.33–1.93	<0.001
Hypertensive disorders	1.30	1.03–1.63	0.027
Oligohydramnios	1.92	1.47–2.50	<0.001
Male fetus	1.44	1.30–1.58	<0.001
Factors Associated with Underestimation of Neonatal Birthweight *			
	Adjusted Odds Ratio	95% CI	*p* Value
Gestational age (every added week)	1.16	1.12–1.20	<0.001

* Adjusted for maternal age, gestational age, nulliparity, obesity (BMI > 30 kg/m^2^), maternal height, diabetes mellitus, smoking, hypertensive disorders, previous cesarean deliveries, oligohydramnios, fetal sex and placental location.

**Table 4:** Multivariable logistic regression analysis—Factors associated with maternal or neonatal adverse outcomes.

Increased Risk for 3rd-or 4th-Degree Perineal Lacerations (Adjusted for Diabetes Mellitus, Obesity (BMI > 30 kg/m^2^), Fetal Macrosomia)	Adjusted Odds Ratio	95% CI	*p* Value
Underestimation of fetal birthweight	1.59	1.05–2.43	0.030
Nulliparity	3.37	2.37–4.80	<0.001
**Increased Risk for Shoulder Dystocia** (adjusted for obesity (BMI > 30 kg/m^2^))	**Adjusted Odds Ratio**	**95% CI**	***p* Value**
Underestimation of fetal birthweight	1.61	1.05–2.46	0.030
Diabetes mellitus	2.06	1.32–3.22	0.002
Nulliparity	3.37	2.37–4.80	<0.001
Fetal macrosomia	5.35	3.45–8.32	<0.001
Increased Risk for Neonatal Composite Adverse Outcome * (adjusted for gestational age and neonatal birthweight)	**Adjusted Odds Ratio**	**95% CI**	***p* Value**
Overestimation of fetal birthweight	1.15	1.02–1.30	0.023
Nulliparity	1.61	1.46–1.76	<0.001
**Increased Risk for Induction of Labor**	**Adjusted Odds Ratio**	**95% CI**	***p* Value**
Overestimation of fetal birthweight	1.30	1.21–1.40	<0.001
Gestational age at delivery (every added week)	1.25	1.22–1.28	<0.001
Nulliparity	1.93	1.83–2.04	<0.001
Diabetes mellitus	2.10	1.94–2.27	<0.001
Hypertensive disorders	4.57	3.98–5.25	<0.001
Neonatal birthweight (every added kg)	1.34	1.24–1.46	<0.001
**Increased Risk for Cesarean Delivery** (adjusted for hypertensive disorders and gestational age)	**Adjusted Odds Ratio**	**95% CI**	***p* Value**
Overestimation of fetal birthweight	1.59	1.41–1.79	<0.001
Nulliparity	1.78	1.63–1.95	<0.001
Diabetes mellitus	1.41	1.24–1.60	<0.001
Maternal obesity (BMI >30 kg/m^2^)	1.44	1.27–1.63	<0.001
Neonatal birthweight (every added kg)	1.48	1.29–1.70	<0.001

* Including hypoglycemia, hypothermia, meconium aspiration, non-invasive ventilation, phototherapy.

## Data Availability

Data will be made available from the corresponding author upon reasonable request.

## Abbreviations

EFW	Clinically estimated fetal birth weight
BW	Birth weight
GA	Gestational age
VE	Vacuum extraction
IOL	Induction of labor
CD	Cesarean delivery
BMI	Body mass index
DM	Diabetes mellitus
GDM	Gestational diabetes mellitus
SGA	Small for gestational age
LGA	Large for gestational age
Aga	Average for gestational age
OR	Adjusted odds ratio
CI	95% confidence interval
